# Rise in Serum 25-Hydroxyvitamin D Levels during the COVID-19 Pandemic

**DOI:** 10.3390/nu16152449

**Published:** 2024-07-27

**Authors:** Witt Durden, Shady Ezaldin, Jessica Amos, Suzanne Kemper, James Campbell

**Affiliations:** 1 Charleston Area Medical Center, Institute for Academic Medicine, 3200 MacCorkle Ave SE, Charleston, WV 25304, USA; shady.ezaldin@vandaliahealth.org (S.E.); jessica.amos@vandaliahealth.org (J.A.); suzanne.kemper@vandaliahealth.org (S.K.); james.campbell@vandaliahealth.org (J.C.); 2 Department of Internal Medicine, West Virginia University-Charleston Division, Charleston, WV 25304, USA

**Keywords:** COVID-19, serum 25-hydroxyvitamin D, vitamin D, hypervitaminosis D

## Abstract

With the emergence of the COVID-19 pandemic, the absence of established evidence-based treatment protocols led healthcare professionals and the public to explore experimental treatments, including high doses of vitamin D supplements. This study aimed to assess changes in serum 25-hydroxyvitamin D levels during the pandemic, employing a retrospective cohort analysis of data from Charleston Area Medical Center (CAMC). The study analyzed serum 25-hydroxyvitamin D levels in a cohort of 35,556 patients treated at CAMC in 2019, a representative pre-pandemic period, to 2021, a representative pandemic period. Our findings revealed a significant increase in mean serum 25-hydroxyvitamin D levels as compared with 2019 (37 ± 21 vs. 31 ± 15 ng/mL, *p* ≤ 0.001). Additionally, in 2021, there were significantly more patients exhibiting serum levels in the highest quintiles, specifically >100 ng/mL (1.6% vs. 0.2%), 75–100 ng/mL (4.6% vs. 1.2%), and 50–75 ng/mL (16.0% vs. 8.4%), *p* ≤ 0.001. This upsurge suggests increased intake of vitamin D supplements, potentially fueled by widespread discussions that were taking place largely on the internet regarding the efficacy of vitamin D against COVID-19. Our findings underscore the critical need for evidence-based public health messaging, especially during health crises, to prevent unnecessary health risks and ensure patient safety.

## 1. Introduction

The beginning of the COVID-19 pandemic brought uncertainty and fear secondary to the emergence of a novel virus. With the lack of evidence-based treatment options, both patients and clinicians alike were willing to try experimental and unproven treatments. Treatment and prevention strategies, ranging from prescription medications, such as hydroxychloroquine, to over-the-counter vitamin supplements, such as vitamin D, were being disseminated on the internet [1,2]. Additionally, due to the urgency of developing effective treatments, many studies were performed that suggested positive outcomes. Most of these trials were observational with confounding variables, and practice-changing recommendations based on these studies were limited [3,4,5]. One of the earliest of these yet-to-be-proven treatments was the use of high-dose vitamin D supplementation to protect against severe COVID-19 disease.

Over the past decade, an extra-skeletal role for vitamin D has been identified. There is credible evidence that vitamin D is an immuno-modulator with effects on inflammation and immunity [6,7]. A small but significant protective effect of vitamin D supplementation in the prevention of acute respiratory infections has been suggested by prior studies [8]. The potential role of vitamin D in treating or preventing COVID-19 was the subject of extensive research and numerous clinical trials after the pandemic’s onset. In 2020 alone, there were several observational studies suggesting that vitamin D levels were inversely correlated with the severity of COVID-19 infection [9,10]. Many of these studies used serum 25-hydroxyvitamin D [25(OH)D] levels at the time of hospital admission, while others compared levels as many as 10–14 years prior to the arrival of COVID-19. These results led to controlled trials that concluded mixed results likely confounded by differences in the study populations and vitamin D supplementation regimens [8,11,12]. More recent studies have suggested protective effects of vitamin D supplementation against COVID-19 infection, but the clinical outcomes examined did not include mortality or the rate of ICU admission [13,14,15,16]. It also has been demonstrated that serum 25(OH)D levels decreased rapidly in acute COVID-19 infections of patients who were hospitalized [17]. This makes assigning causality difficult.

Even though there was limited direct evidence that vitamin D protects against COVID-19, it was still used extensively in 2021 in both small and large doses of up to 60,000 IU per day. As a result, higher serum 25(OH)D levels were noted by physicians in clinical practice, raising concerns about possible vitamin D misuse and potential hazards [17,18]. One study in Ireland showed the average yearly serum 25(OH)D measurement increased by 2.8 nmol/L in the first year after COVID-19, which was almost threefold higher than two similar trend analyses that were conducted between 1993 and 2016 [19].

To our knowledge, there have been no studies carried out in the United States thus far to evaluate average serum 25(OH)D levels and potential vitamin D misuse as a result of the COVID-19 pandemic. We examined serum 25(OH)D levels in a patient cohort defined by care location to observe whether COVID-19 fears in the United States led to vitamin D supplementation and potential vitamin D misuse.

## 2. Materials and Methods

### 2.1. Study Population

This was a retrospective cohort study of patients 18 years or older using the electronic health records (Cerner) of Charleston Area Medical Center (CAMC). Both inpatients and outpatients were included in the analysis. We extracted demographic data and serum 25(OH)D levels from all patients treated at CAMC from either 1 January 2019–31 December 2019, representing the pre-pandemic period, or 1 January 2021–31 December 2021, the pandemic period. The serum 25(OH)D measurements were collected as part of routine screening utilizing a Chemiluminescent Immunoassay (CLIA) following the Beckman Coulter quality control scheme every 24 h.

### 2.2. Quantitation of Serum 25-Hydroxyvitamin D

CAMC labs, which maintain CAP (College of American Pathology) and CLIA (Clinical Laboratory Improvement Amendments) accreditation, performed serum testing for 25(OH)D. The current assay for 25(OH)D at CAMC labs is the Beckman Coulter DxH 900 assay. Prior to July of 2020, the analysis was performed on the DiaSorin Liaison XL device. Both tests utilize an automated chemiluminescence immunoassay methodology.

### 2.3. Statistical Analysis

After data extraction into Excel, SAS 9.4 (SAS Institute Inc., Cary, NC, USA) was used to generate summary statistics and comparisons for variables of interest. With a sample larger than 2000, the Kolmogorov–Smirnov nonparametric test was computed for normality testing. Results showed data were not normally distributed as the Kolmogorov–Smirnov test was significant (*p* < 0.01). Nonparametric tests performed included Mann–Whitney U and Kruskal–Wallis. A *p* < 0.05 was considered statistically significant.

## 3. Results

A total of 35,556 patients were included in the study. Their average age was 54.1 ± 20.7 years, 62.5% were females, and 93.5% were Caucasian. Most patients were seen as outpatients (*n* = 31,148, 87.6%). Approximately 10% were cared for as inpatients (*n* = 3784, 10.6%), and the remaining 1.8% (*n* = 624) were treated in the emergency department, considered with observation, or were patients seen more than two times.

Overall, the mean serum 25(OH)D levels for the cohort were 35 ± 19. When the patients were split into quintiles based on serum 25(OH)D levels, most patients had serum 25(OH)D levels that were considered within normal clinical ranges: <25 ng/mL (*n* = 11,687, 32.9%), 25–50 ng/mL (*n* = 17,866, 50.3%), 50–75 ng/mL (*n* = 4528, 12.7%), 75–100 ng/mL (*n* = 1129, 3.2%), and >100 ng/mL (*n* = 346, 1.0%), (Table 1). Overall, females had higher mean serum 25(OH)D levels than males (35 vs. 34, *p* < 0.001) (Table 1). The mean serum 25(OH)D levels in other race groups were significantly lower than the mean serum 25(OH)D levels in Caucasians (29 vs. 35, *p* < 0.001) (Table 1).

The patients seen at CAMC in 2021 were older on average than the patients seen in 2019 (mean 54.3 ± 20.0 vs. 53.8 ± 21.5 years, *p* = 0.03), and more patients were seen by the healthcare system in 2021 (*n* = 15,318 in 2019, 43% of the cohort vs. *n* = 20,238 in 2021, 57% of the cohort). Mean serum 25(OH)D levels were significantly higher in 2021 than in 2019 (37 ± 21 vs. 31 ± 15 ng/mL, *p* ≤ 0.001). When assessed by quintiles, significantly more patients exhibited serum 25(OH)D levels in the higher ranges in 2021 than in 2019 ((*p* ≤ 0.001): >100 ng/mL (1.6% vs. 0.2%), 75–100 ng/mL (4.6% vs. 1.2%), and 50–75 ng/mL (16.0% vs. 8.4%) (*p* = ≤0.001). Conversely, fewer patients were in the lowest serum 25(OH)D level quintiles in 2021 as compared with 2019: 25–50 ng/mL (52.2% vs. 48.7%) and >25 ng/mL (37.9% vs. 29.0%) (Table 2).

**Figure 1:**
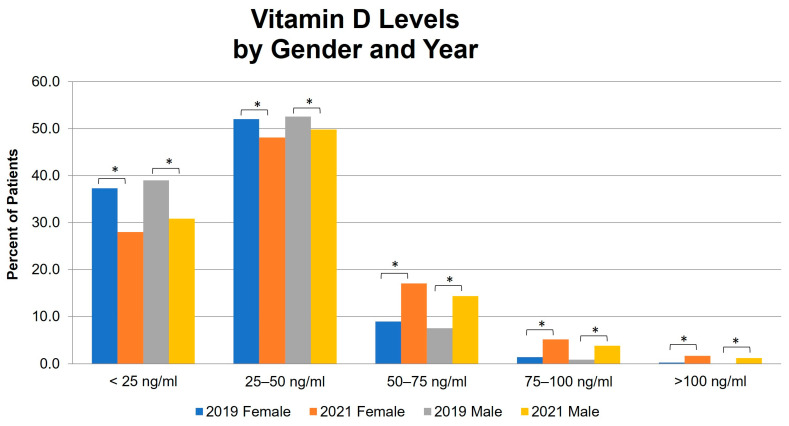
Serum 25-hydroxyvitamin D levels stratified by quintile, gender, and year. * *p* ≤ 0.001.

When the patients were stratified by both gender and serum 25(OH)D levels, a significant increase was observed in 2021 in the proportion of both males and females with serum 25(OH)D levels ≥ 50 mg/mL as compared with 2019 (Figure 1).

## 4. Discussion

Our study showed an increase in the mean serum 25(OH)D in adult patients during the height of the pandemic as compared with the year prior to the appearance of COVID-19 in the United States. We also observed a significant increase in the number of patients with serum 25(OH)D levels at and above the upper level of normal (>50–100 ng/mL) during the pandemic. The level at which serum 25(OH)D becomes toxic is not well defined, but 80 to 100 ng/mL is generally considered as the upper limit of normal [20]. The commonly used threshold for excess serum 25(OH)D is defined as >100 ng/mL, while intoxication is >150 ng/mL [21]. The lab assay that was employed at our institution only reported >120 ng/mL due to quality control measures and thus cannot comment specifically on the number who would have crossed the threshold of 150 ng/mL. As levels rise above the upper limit of normal, the potential risk for adverse effects increases [22]. One randomized trial showed decreased rates of acquiring COVID-19 infection in healthcare workers who took vitamin D supplementation. However, like much of the data early in the pandemic, it is difficult to make conclusions based on this study alone due to the small numbers and limitations of the study (i.e., the high dropout rate of those with baseline vitamin D deficiency) [23]. Despite great interest in vitamin D supplementation for various indications, there is still uncertainty regarding its benefits and safety, especially in patients with adequate serum 25(OH)D levels prior to supplementation [4,24,25,26].

The most likely explanation for our findings is that patients were taking more vitamin D supplements during the year 2021 than in 2019. Natural vitamin D synthesis through sunlight will not raise serum 25(OH)D levels above the upper limit of normal due to feedback mechanisms [27,28,29,30]. Thus, the only way to reach levels above 100 ng/mL is through supplementation. The suggestion that vitamin D could treat or prevent severe COVID-19 infection likely led to this supplementation.

Observational and small randomized trials early in the pandemic suggested that vitamin D supplementation might lower mortality and protect against severe COVID-19 infection [30,31,32,33]. Unfortunately, the level of attention that some studies received did not necessarily correspond with their quality. For example, one study, which had yet to be peer reviewed and which has since been removed from the online repository where it appeared, led to thousands of Twitter posts and even “headlines in major news outlets” [24]. An analysis of YouTube videos during this time also revealed nearly three-quarters of the videos included misleading information about COVID-19 and almost 80% contained misleading information about vitamin D [2]. Because the only way to have supratherapeutic serum 25(OH)D levels is through dietary supplementation, our study suggests a temporal association between a marked spike in serum 25(OH)D levels and the claims of vitamin D benefits on the internet, some of which were misleading or weakly supported by the available clinical data.

A possible limitation of our study is the fact that serum 25(OH)D levels were checked in more patients during the midst of the pandemic (2021) than the year prior to the pandemic, which could account for the greater number of high values. However, this further implies that vitamin D was of increased interest to both providers and patients during this period. The small proportion of non-Caucasian patients cared for at our center, and therefore available for our study, is another limitation. The baseline serum 25(OH)D levels were lower in non-Caucasian patients prior to the pandemic, but levels rose similarly during the pandemic. The applicability of the findings to a more diverse population will require further investigation.

## 5. Conclusions

In this study, we observed a significant increase in serum 25(OH)D levels during the COVID-19 pandemic as compared with the pre-pandemic period. The observed, population-wide increase included an increase in the proportion of patients with hypervitaminosis D. The rise in serum 25(OH)D levels coincided with widespread reports regarding its potential benefits in treating COVID-19. This temporal association suggests a link to these claims, despite conflicting evidence supporting them at the time.

This study highlights the need for evidence-based information dissemination during public health emergencies to ensure patient safety.

## Figures and Tables

**Table 1:** Overall demographics and serum 25-hydroxyvitamin D quintiles.

	Total Patients *n* (%) ^1^	Mean Serum 25(OH)D, ng/mL	*p* Value
All patients	35,556 (100%)	35 ± 19	
Age, mean years ± SD	54.1 ± 20.7		
**Gender** ^2^			≤0.001
Female	22,203 (62.5%)	35 ± 19	
Male	13,346 (37.5%)	34 ± 18	
**Race** ^3^			≤0.001
White	31,618 (93.5%)	35 ± 19	
Other ^4^	2200 (6.5%)	29 ± 19	
**Serum 25(OH)D quintiles**			≤0.001
<25 ng/mL	11,687 (32.9%)	18 ± 5	
25–50 ng/mL	17,866 (50.3%)	35 ± 7	
50–75 ng/mL	4528 (12.7%)	59 ± 7	
75–100 ng/mL	1129 (3.2%)	84 ± 7	
>100 ng/mL	346 (1.0%)	112 ± 9	

^1^ Unless otherwise indicated. ^2^ Available for 35,549 patients. ^3^ Available for 33,818 patients. ^4^ Other included American Indian or Alaska Native, Asian, Black or African American, Native Hawaiian or Pacific Islander, and multiple races documented.

**Table 2:** Serum 25-hydroxyvitamin D levels prior to and during the COVID-19 pandemic.

	2019 (*n* = 15,318) *n* (%) ^1^	2021 (*n* = 20,238) *n* (%) ^1^	*p* Value
Age, mean years ± SD	53.8 ± 21.5	54.3 ± 20.0	0.03
**Gender** ^2^			0.6
Female	9587 (62.6%)	7621 (37.7%)	
Male	5725 (37.4%)	12,616 (62.3%)	
**Race** ^3^			0.17
White	13,816 (93.7%)	17,802 (93.3%)	
Other ^4^	928 (6.3%)	1272 (6.7%)	
**Serum 25(OH)D, mean ng/mL ± SD**	31 ± 15	37 ± 21	≤0.001
**Serum 25(OH)D quintile**			≤0.001
<25 ng/mL	5809 (37.9%)	5878 (29.0%)	≤0.001
25–50 ng/mL	8001 (52.2%)	9865 (48.7%)	≤0.001
50–75 ng/mL	1287 (8.4%)	3241 (16.0%)	≤0.001
75–100 ng/mL	189 (1.2%)	940 (4.6%)	≤0.001
>100 ng/mL	32 (0.2%)	314 (1.6%)	≤0.001

^1^ Unless otherwise indicated. ^2^ Available for 35,549 patients. ^3^ Available for 33,818 patients. ^4^ Other included American Indian or Alaska Native, Asian, Black or African American, Native Hawaiian or Pacific Islander, and Multiple races documented.

## Data Availability

The raw data supporting the conclusions of this article can be made available by the authors on request.
